# An overview of a preliminary multicenter retrospective study on food and drug allergies in Moroccan pediatric population

**DOI:** 10.11604/pamj.2024.47.24.41038

**Published:** 2024-01-22

**Authors:** Zakaria Zidane, Chaimaa Chahine, Karima Mohtadi, Azeddine Chakroun, Rachid Saïle, Ahmed Aziz Bousfiha, Maria Rkain, Sanae Elimlahi Chaer, Youness El Gueddari, Mohammed Hbibi, Laila Tazi Daoudi, Ibtihal Benhsaien, Naima Elhafidi, Hanane Salih Alj

**Affiliations:** 1 Laboratory of Biology and Health, Research Center of Biotechnologies and Health, University Hassan II of Casablanca, Faculty of Sciences Ben M´sik, Avenue Cdt Driss El Harti, B.P 7955, Sidi Othmane, Casablanca, Morocco; 2 Laboratory of Clinical Immunology, Inflammation and Allergies, University Hassan II of Casablanca, Faculty of Medicine, 19 Rue Tarik Ibnou Ziad, BP 9154, Casablanca, Morocco; 3 Pediatric Service, University Hospital Mohammed VI, Oujda, Morocco; 4 Liberal Sector Allergologists, Casablanca, Rabat and Oujda, Morocco,; 5 Pediatric Service, University Hospital Hassan II, Fès, Morocco; 6 Laboratory of Medical Biotechnology, University of Mohammed V de Rabat, Faculty of Medicine of Rabat, Impasse Souissi, Rabat 10100, Morocco

**Keywords:** Food, drug, allergies, survey, management, Morocco, epidemiology

## Abstract

**Introduction:**

this study aimed to investigate the prevalence and management of food allergies (FA) and drug allergies (DA) in Morocco. Sparse and conflicting epidemiological data exist on the exact prevalence of allergies in the country. The rise in allergies can be attributed to various factors.

**Methods:**

the study analyzed data from patients with suspected FA and DA who sought medical attention. Statistical tests were used to analyze the data, percentages were computed for qualitative variables, and for quantitative variables, medians or means accompanied by standard deviations (SD) were calculated. The Chi-square test was employed to assess categorical variables. A p-value < 0.05 was considered statistically significant.

**Results:**

Cow's milk was the most reported food allergen (58.2%), followed by egg and nuts (23.4% and 12.1%, respectively). The most affected age group was children under 5 years. Antibiotics were the leading cause of reported drug allergies (44.8%), particularly Beta-lactams. Immediate reactions were commonly associated with antibiotics and nonsteroidal anti-inflammatory drugs (NSAIDs). Symptoms of FA included acute urticaria, vomiting, anaphylactic shock, and facial edema. Urticaria was the most frequent symptom of DA. Antihistamines and corticosteroids were the main treatments used for both FA and DA.

**Conclusion:**

the prevalence of FA and DA in Morocco remains uncertain due to limited data. There is a need for centralized data collection and awareness among clinicians and the general population regarding allergies. The study highlights the importance of proper diagnosis and management of allergies to ensure patient safety. The findings emphasize the necessity of establishing a mandatory center for allergy care in Morocco to improve the understanding and management of allergic conditions.

## Introduction

Allergies are a common condition that has considerable impact on public health. It represents an important part of chronic diseases [1], affecting more than 2% of populations in most developed countries [2]. Prevalence of allergies continues to increase worldwide. World Allergy Organization (WAO) estimates it between 10 and 40% in the general population according to concerned countries [2,3]. In the same way, World Health Organization (WHO) considers allergies as the 4^th^ largest chronic health issue in the world, leading to serious risk of pandemic situation. In the new global economy, allergies have become a central issue involving direct costs (consultation, hospitalization, medication) and indirect ones (decrease of life quality, absenteeism and reduced performance) [4,5].

One of the main obstacles concerning this issue is heterogeneity in studies worldwide, which makes its epidemiology, diagnosis and management not all well known. WAO remains the only alliance of allergology and clinical immunology society worldwide [6], it provides allergy work-ups, updates and surveys. It brings together more than 15 societies (e.g. American Academy of Allergy, Asthma and Immunology, AAAAI; European Academy of Allergy, Asthma and Clinical Immunology, EAACI; Japanese Society of Pediatric Allergy and Clinical Immunology, JSPACI; Australasian Society of Clinical Immunology and Allergy, ASCIA...). Considering the importance of this issue in public health, it is fundamental that allergy definition have to be as precise as possible. Indeed, hypersensitivity is an abnormal and excessive response to an exogenous antigen. Depending on the mechanism, hypersensitivity can be grouped into three general types [7]: a) allergic immunological hypersensitivity (HSIA), which is based on an immunological function mechanism, dependent on adaptive immunity receptors (B-cell receptor (BCR) or T-cell receptor (TCR); b) non-allergic immunological hypersensitivity (HSINA), based on an antigen-non-specific innate immunity mechanism; c) non-immunological hypersensitivity (HSNI), involving toxic, enzymatic or pharmacological mechanism; in which immune cells aren´t the original target of the antigen. The prevalence of FA is increasing in some regions of the world. However, there is geographical variability in the incidence, type, and clinical presentation of FA as well as variations in symptoms and clinical phenotypes due to race, ethnicity, age, environment and coexisting allergic diseases [8]. The FA affects about 8% of children in the Western countries and seems to be rising in other parts of the world such as in Vietnam and South Africa, and other parts of Asia and Africa, particularly in urban rather than rural areas. Drug hypersensitivities, allergies or drug pseudo allergic reactions represent an important aspect of iatrogenic pathology and the morbidity, mortality and cost associated with these syndromes are often underestimated [9].

There is still a lack of knowledge on DA epidemiology, clinical spectrum, and appropriate diagnostic methods, particularly in children [10]. At the national level, Morocco does not have a structured multidisciplinary network of expertise to address the major issues related to hypersensitivities and to centralize data. Also, doesn´t have investigation centers and prospective declarations, of precise and reliable data on the morbidity of allergic origin to evaluate the impact in the society. A global evaluation would be of high interest for the sustainable control of the morbidity of hypersensitivity. The objective of this study was to initiate a survey on pediatric FA and DA in Morocco. We started from the data available in the four main hospitals and few clinicians concerned and accessible (Casablanca, Rabat, Oujda and Fez), in order to make an inventory to highlight and outline some figures on the epidemiology of FA and DA.

## Methods

**Study setting**: this descriptive survey study was conducted over a period of seven years, from January 1^st^, 2015, to January 2022. The study took place across four university hospitals, namely CHU Ibn Rochd in Casablanca, CHU Ibn Sina in Rabat, CHU Mohammed VI in Oujda, and CHU Hassan II of Fez. Additionally, the corresponding liberal sector data in the mentioned regions was also included. The study aimed to establish and describe the epidemiological, clinical, and therapeutic profile of food and drug allergies in the pediatric population.

**Study participants**: the target population for this study included children (N = 199) under the age of 18 who had experienced type 1 and 4 hypersensitivity reactions related to a suspected food and drug allergen. These allergic reactions encompassed respiratory symptoms, skin rashes, gastrointestinal issues, or anaphylactic reactions. Children with viral rashes or dermatological issues not suggestive of an allergy were excluded to ensure the specificity of the study. The focus was on children with confirmed hypersensitivity reactions to provide a comprehensive understanding of food and drug allergies in the pediatric population.

**Design of the study**: this study utilized a cross-sectional descriptive survey design. It spanned over seven years and aimed to investigate the epidemiological, clinical, and therapeutic aspects of food and drug allergies in pediatric patients. The study employed a systematic random sampling technique to select a representative sample of participants from the target population. Data were collected through a structured questionnaire that had been validated by pediatric immunologists and allergists. The questionnaire consisted of three sections: patient information, clinic-related information, and management and diagnostic procedures.

**Data analysis**: the collected data underwent statistical analysis using the Statistical Package for Social Science (SPSS) version 13.0 for Windows. The analysis involved several statistical methods. Descriptive statistics were used to summarize the demographic characteristics of the study population, such as age and gender. Frequency distributions were employed to analyze categorical variables, including common allergens and clinical manifestations, to identify patterns and associations. Inferential statistical tests, specifically the Chi-squared (Chi2) test, were performed to explore relationships between variables. Statistical significance was considered at ≤0.05.

**Ethical considerations**: this study was conducted in accordance with the principles outlined in the Helsinki Declaration and obtained approval from the regional ethics committee “Medecine and pharmacy faculty of Casablanca/ university hospital center bnou Rochd Casablanca” [N° ordre: 11/2022], to ensure that the study complied with ethical standards and protected the rights and well-being of the participants (Law 28-13, N° 02/DRC/00). Before participating in the study, all legal guardians of participants were required to provide informed consent. Confidentiality of data collected was ensured by using identifiers rather than the names of participants.

## Results

**Figure 1:**
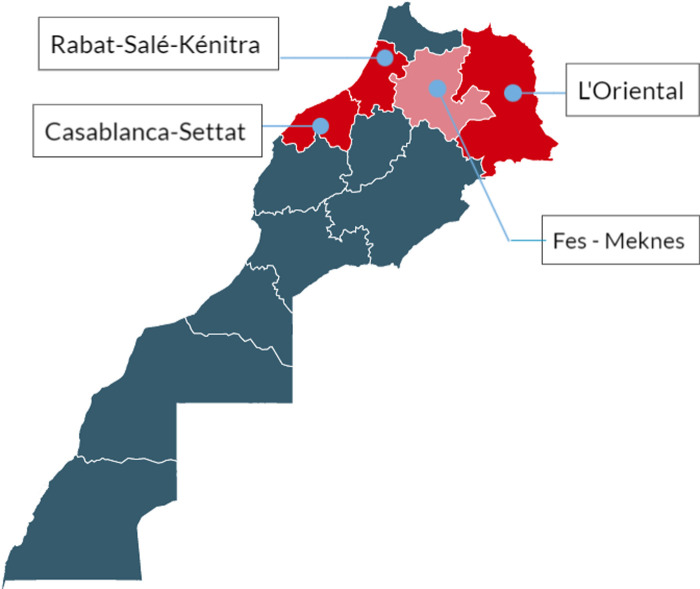
mapping of Morocco showing the 4 regions mentioned in where the survey was performed

Our study lasted 8 years (2015-2022). For FA 141 cases were identified in 4 cities of the kingdom: Casablanca, Rabat, Oujda and Fes (Figure 1). The sample for FA, was composed of 58 girls (41.1%), 78 boys (55.5%) and 5 individuals with not documented gender (3.5%). Concerning DA, 58 cases were reviewed across 3 regions (Casablanca, Rabat, Oujda), patients were composed of 31 boys and 19 girls and gender was not documented for 8 patients.

**Age**: in FA, the mean age is 5.54 years with a minimum of 40 days and a maximum of 18 years. It should be noted that 7 patients have not been documented. The maximum incidence is in the age group under 5.5 years. In DA, the mean age was 7.1 years, with a minimum of 6 months and a maximum of 15 years and it wasn´t documented for nine patients.

**Figure 2:**
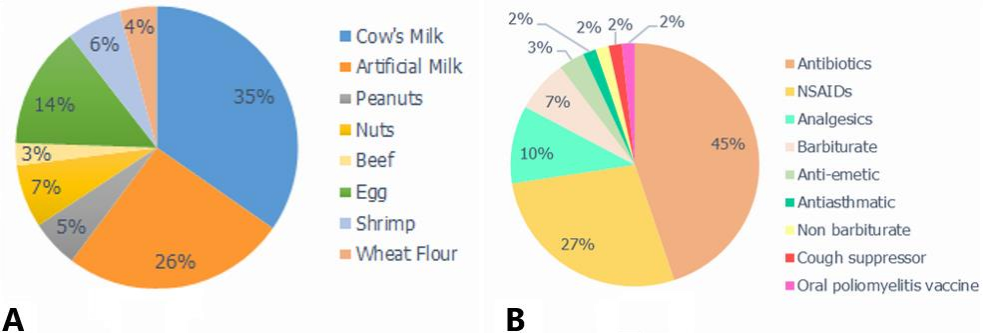
distribution of most incriminated allergens in the population's study: A) showing the distribution of food allergens; B) showing the incriminated allergens by drug classes

**Allergens**: the most food allergens reported (Figure 2A), cow's milk first (58.2%), followed by egg and nuts (23.4%,12.1%) respectively, shrimps (10.6%), peanuts (9.2%), wheat flour (7.1%) then comes artificial milk and beef (4.3%). The most affected patients were in the age group of less than 5 years (Table 1), with a count of 56 cases for cow´s milk allergy, 21 cases for egg allergy and 5 cases for peanut allergy. Of all reported DA cases, 26 were attributed to antibiotics (44.8%) (Figure 2B), which included 17 cases of Beta-lactams allergies. The second most reported class concerns NSAIDs with a total of 16 cases (27.6%). Analgesics, barbiturates and one anti-emetic respectively (10.3%, 6.9% and 3.4%). Finally, an oral poliomyelitis vaccine, an antiasthmatic, a cough suppressor and a non-barbiturate came last with a total of 1 case for each (1.7%).

**Table 1:** distribution of cases by age groups and most incriminated food allergens

Age groups	Cow´s milk allergy	Egg allergy	Peanut allergy
**<5.5**	56	21	5
**5.6-10.5**	17	9	3
**10.6-15.5**	4	1	1
**>15.5**	0	1	3
**Total**	77	32	12

**The main characteristics of food and drug allergy**: the main characteristics of the allergies reported in our study (age groups, main clinical manifestations, time between taking the drug and the reaction, family atopy) and the modalities of management are represented in Table 2 and Table 3.

**Table 2:** main epidemiological and clinical characteristics of reported food allergies

Age groups	Number	Percentage (%)
< 5 years	81	(57,4%)
>5,6 years - 10,5 years	37	(26,2%)
10,6 -15,5 years	11	(7,8 %)
>15,5 years	12	(8,5%)
**Family Atopy**		
Occurence	20	(14,2%)
Not Documented	121	(85,8%)
**Symptoms of the acute phase of the reaction**		
Acute Urticaria	59	(41,9%)
Chronic Urticaria	2	(1,4%)
Atopic dermatitis	11	(11,3%)
Facial Edema	23	(16,3%)
Lips and Tongue Edema	21	(14,9%)
Wheezing Dyspnea	8	(5,7%)
Diarrhea	24	(17%)
Hematochezia	2	(1,4%)
Vomiting	42	(29,8%)
Rhinitis	10	(7,1%)
Anaphylactic shock	41	(29,1%)
GOR	12	(8,5%)
Other*	75	(53,2%)
**Treatment of the acute phase of the reaction**		
Antihistamines	75	(53,2%)
Corticosteroids	64	(45,4%)
Adrenalin	26	(18,4%)
Allergen avoidance	52	(36,9%)
Other**	70	(49,6%)

* The other manifestations are isolated symptoms or the combination of several symptoms (cough, fever, eczema, celiac, etc.) which are typical and/or atypical of allergies, and which may be influenced by other physiological factors. ** For the 49.6% of the other cases, other treatments were prescribed such as antidiarrhea, PPIs and, in a few cases, hydrolyzed rice or soy milk instead of artificial milk or cow's milk.

**Table 3:** main epidemiological and clinical characteristics of reported drug allergies

Reported Cases		
**Age groups**	**Number**	**percentage**
6 months-6 years	23	(39.7%)
> 6 years -12 years	17	(29.3%)
> 12 years	9	(15.5 %)
Not Documented	9	(15.5%)
**Family Atopy**		
Occurrence	15	(25.9%)
Not Documented	1	(1.7%)
**Symptoms of the acute phase of the reaction** (n=158)		
Acute Urticaria	28	(48.3%)
Facial Edema	21	(36.2%)
Lips and Tongue Edema	16	(27.6%)
Wheezing Dyspnea	11	(19%)
Diarrhea	11	(19%)
Vomiting	13	(22.4%)
Rhinitis	5	(8.6%)
Conjunctivitis	5	(8.6%)
Severe Toxidermia	14	(24.1%)
Other*	34	(58.6%)
**Time between drug use and reaction**		
Immediate	39	(67.2%)
Delayed	15	(25.9%)
Not Documented	4	(6.9%)
**Treatment of the acute phase of the reaction**		
Antihistamines	49	(84.5%)
Corticosteroids	44	(75.9%)
Epinephrine	11	(19%)
Discontinuing of SD	31	(53.4%)
Avoidance of SD	29	(50%)
Other**	18	(31%)

* Other manifestations are isolated symptoms or combination of several symptoms (cough, cyanosis, fever, cheilitis…) which are typical and/or atypical of allergies, and which may be influenced by the nature of the suspected drug, the route of administration, and the combination of different drugs or by other physiological factors. ** For the remaining 31% of cases, additional treatments for toxidermia, contraindication of skin tests and other treatments for associated symptoms (antipyretics, antiemetics...) were prescribed and indicated.

**Clinical profile**: in FA, the symptomatologic profile included acute phase of the reaction, the profile was dominated by: Acute urticaria in 59 cases (41,9%) (Table 2), followed by vomiting in 42 cases (29,8%), anaphylactic shock in 41(29.1%), then facial edema in 23 cases (16. 3%). For DA, urticaria is the most frequent (48,3%) (Table 3) facial edema, lips and tongue edema were the second most frequent manifestations (36% and 27,6% respectively). Severe Toxidermia ranked 3^rd^with a frequency of 14 cases (24,1%), 3 of which developed the most severe form (Lyell´s disease).

**Management of the reaction's acute phase**: treatment with antihistamines (AntiH1), corticosteroid therapy was first-line treatment in FA and are administered in almost all cases (53.2%, 45.5%, 36.9% respectively) (Table 2). Adrenaline was administered in 18,4% of cases when showed signs of anaphylactic shock and severe edema such as angioedema. The reactions for DA were treated mainly with AntiH1 and corticosteroids, and were administered in almost all cases (84.5% and 75.9% respectively) (Table 3). Discontinuation and avoidance of suspected drugs were indicated for 53.4% and 50% of patients respectively. Epinephrine was administered for 19% of the cases all with signs of shock and severe edema such as angioedema.

**Figure 3:**
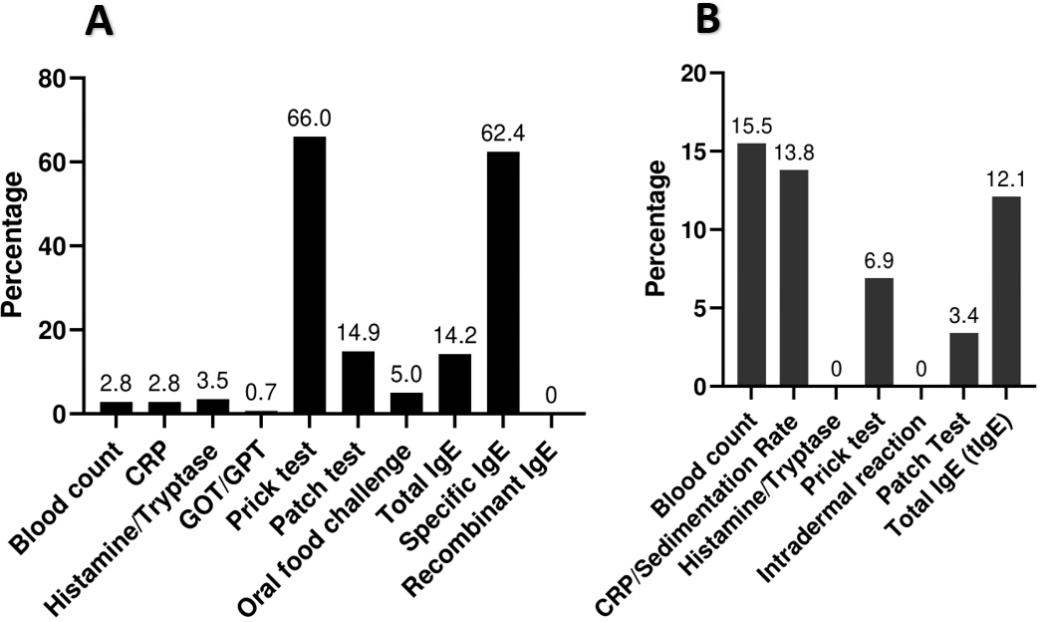
distribution of cases according to specific allergy tests performed; (A) for food allergy and (B) for drug allergy

**Diagnosis profile**: the most used FA tests, we found the prick tests that were carried out in 93 patients (66%) (Figure 3A), the dosage of specific IgE was carried out in 88 patients (62.4%) then patch tests which were carried out in 21 patients (14.9%). Food challenge was performed in 7 patients (5%) all with cow's milk always negative results up to the dose of 50 ml. For DA diagnosis, there were low rates of allergological tests performed (Figure 3B). Total IgE tests were performed for 12.1% of patients, followed by prick tests for 4 patients. Patch-tests were performed for 2 patients with delayed and potentially severe reactions. A significant difference was observed for the type of reaction (Immediate/Delayed) by drug class (p=0.01). Antibiotics and NSAIDs were most frequently responsible for immediate reactions (46.2% and 33.3% respectively). Reported cases in DA were predominantly males (53.4%), (p=0,0001).

## Discussion

Of all the patients present in our study 77 cases (Table 1) had an IgE-mediated cow's milk allergy, and cow´s milk was the most incriminated allergen (58.2%). We found a persistence of cow´s milk allergy in the age group under 5 years (Table 1) and these results are consistent with several studies [3-11]. This allergy is fixed in children from birth to two and a half years, and it should be noted that food allergens of animal origin predominate in children up to eight years of age, while those of plant origin are more frequent in adolescents and adults [12]. In a study of a population of 6209 neonates, it was found that the supplementation of breastfeeding with a cow's milk formula during the first three days of life in maternity favors the appearance of an allergy to cow's milk protein upon subsequent re-exposure to cow's milk [13]. A recent study in 2021 conducted in France by (Tamazouzt *et al*.) [14], cow´s milk was the most often found allergen, followed by eggs and then peanuts, and among the children participating in the “Europrevall” research project, (23.6%) had a cow's milk allergy [15]. The predominance can be explained by the fact that these bovine proteins are the first ingested by infants [12] and it is noted that the allergy to cow's milk always manifests itself during the first year of life of an infant, because at this age the newborn is exposed very early to antigenic stimulation, as well as its intestinal mucosa is very permeable to antigenic macromolecules due to digestive and immunological immaturity [11].

We found that in second place comes egg allergy with 32 cases (Table 1 and a percentage of 23.4% (Figure 2A) as the most incriminated allergen after cow's milk, and our results are consistent with a recent US study conducted in 2020, where they found that egg allergy is the second food allergy after cow's milk in young children [16]. Eggs are the most allergenic food in children under three years old, the egg white (ovalbumin) is more allergenic than egg yolk [11]. As a general rule, egg allergy appears with the ingestion of products containing egg proteins, other triggering pathways are possible, such as simple skin and/or mucosal contact with egg proteins [17]. Then we found that peanut allergy comes in third place with 12 cases (Table 1) and these results are consistent with a study conducted in France [18,19]. Peanut allergies can appear early in life, between the ages of two and three. Early sensitizations have been described in infants who have never consumed peanuts. The children were sensitized in utero or via breast milk, the possibility of sensitization by peanut oil, present in certain medicinal preparations, has also been reported [20]. The prevalence of cow´s milk allergy is estimated at (0.7%) on the mean age in Europe, in the Europrevall birth cohort, the mean age incidence of egg allergy was estimated at (1.3%). In turn, the prevalence of peanut allergy in children will double in the space of ten years in the United Kingdom, north America and Australia. it represents approximately 1.8%, 1.4% and 3% respectively [3]. For other countries such as Asia, south America, central America and Africa, FA is considered rare and epidemiological data are limited [21-23]. As the case in our country, Morocco represents an unexplored sector in terms of data concerning the allergic complaint. The EAACI algorithm for diagnosing FA includes five critical steps [8]: (1) patient history with structured questions, (2) sensitization assessment using standardized SPT and/or sIgE, (3) diagnostic elimination diet i.e. short-term avoidance (4) the OFC to definitely confirm or exclude the diagnosis (5) assessment of non-IgE-mediated FA if history is convincing and SPT results and sIgE are negative. One of the challenges of FA diagnosis in some countries, that they have limited resources, in terms of the number of trained “allergist” specialists as well as access to “SPT” reagents in laboratories, and some countries do not currently have an accredited national program for the training of specialists in allergology [21,24].

It can be noted that the same is true for Africa and more specifically in our country, where the absence of documents on the topic of allergies in Morocco, and this point must be considered a priority for the need for care patients in both the private and public sectors. All of the diagnostic tests mentioned above do not always allow easy identification of the allergen in question, with sometimes questionable or even contradictory results with clinical results. It is in this context that other examinations exploring immediate hypersensitivity reactions are sometimes carried out [3]. Although recent advances in diagnostics (e.g., basophil activation assays) hold great promise for improving the accuracy and reliability of food allergy diagnosis since the OFC is impractical and although their availability is limited worldwide and the technical requirements of laboratories make them of little use in many clinical and epidemiological settings [25]. The main management presented in our study for food allergy is based on the eviction of food or foods containing allergenic substances. In addition to avoidance, the symptoms can be relieved by local or systemic treatments, the main treatments of which are antihistamines which block the action of histamine released by the inflammatory cells during the allergic reaction are often prescribed to relieve the symptoms of urticaria, rhinitis, or even allergic conjunctivitis [8,26].

The corticosteroids which are prescribed in the case of eczema, then finally adrenaline which is an emergency treatment in case of anaphylactic shock. In the form of an auto-injector, it has an action on the heart and blood vessels which leads to the proper functioning of blood circulation. Most organizations around the world such as the EAACI and the AAAA also recommend complete and permanent avoidance of the offending allergen in the first place. As with drug treatment, antihistamines sometimes reduce skin symptoms and oral syndrome [8]. The appearance of oral immunotherapy (OIT) using food products allowing the ingestion of the allergen is increasingly widespread in private practices in the United States. without forgetting that in January 2020 Food and drug administration (FDA) approved “Palforzia” which is the first drug for FA for the treatment of children from 4 to 17 years old suffering from peanut allergy, then in December 2020, the European Medicines Agency (EMA) also approved it [8]. For DA, the number of cases affected is relatively small compared to the expected number. This may be due, on the one hand, to the fact that all patients are subjected to an emergency consultation and/or hospitalization after the onset of clinical manifestations, and not for a consultation for the purpose of exploring an allergy. On the other hand, decentralization of data has potentially been a factor that has led to the identification of this relatively small number. The rare occurrence of DA in pediatric patients can also explain this low rate of our study [27]. Epidemiological data on drug allergies are very imprecise and scarce and therefore cannot be compared to bibliography [28].

For drug classes, the majority of reported allergies involve antibiotics, particularly Beta-lactams. These results are consistent with the overall literature published to date. A retrospective study of Piccorossi *et al*. [29] on drugs, antibiotics with the majority of Beta-lactams and NSAIDs dominating the allergenic profile. Rubio *et al*. [30] finds that these antibiotics (Beta-lactams first) and NSAIDs are the most incriminated drugs in children. Similarly, in a retrospective series of Katsogiannou *et al*. [31], antibiotics of which 64.93% of Beta-lactams, followed by NSAIDs are the most common. This distribution of drug classes, apart from any consideration of the really allergic nature of the reactions, is consistent with the frequency of prescription of these drug classes in ambulatory or hospital medicine, or by self- medication, where the diagnosis of infections is frequent.

Unexpectedly, our study found a relatively low number of family atopy. Genetics alone cannot be a major risk factor for allergies, but also long-term exposure to allergens can be responsible for the decrease in family atopy. Some elements related to lifestyle, family, social or geographical environment could also play a role in the appearance of personal atopy and decrease of family atopy [32]. The clinical pattern and factors contributing to allergic responses were in line with results from prior studies [33,34]. Cutaneous eruptions were the most common symptoms. For example, Erkoçoğlu *et al* [35], finds that the cutaneous eruption is the most frequent symptoms in DA. In our study, the diagnosis of DA is almost not made for almost all patients in our series. Unlike many studies that opt for a complete allergy work-up [4]. This observation can be explained by several reasons (difficulty in establishing a specific allergy diagnosis for public institutions, very high cost of skin and in-vitro tests, unavailability of specific IgEs of all drug molecules).

The use of skin tests such as skin prick-tests (SPT) and intradermal tests (IDT) in cases of acute reactions and for some medicines is advised by current guidelines, however it may be challenging for this population [36,37]. IDT can be difficult for young children to handle since they are poorly standardized, logistically challenging and relatively painful [36-38]. For non-immediate reactions, such as exanthema that appear hours to days after drug administration, drug provocation tests (DPT) without preceding skin testing have been suggested [39]. Children are frequently wrongly excluded access to effective medication when a complete allergy work-up is not performed at all or performed after a very long period following the likely beginning of drug reaction [40]. However, even though the majority of adverse events in children are thought to be allergic, only a small number of suspected reactions are verified following a prompt comprehensive allergy work-up.

**Limitations**: limited sample size: the study was conducted in four specific hospitals and regions in Morocco, which may not fully represent the entire pediatric population of the country. The sample size may be small, limiting the generalizability of the findings. Lack of comprehensive data: The study relied on available data from hospitals and clinicians, which may not capture the complete picture of food and drug allergies in Morocco. The absence of a structured multidisciplinary network and centralized data collection hindered obtaining precise and reliable information on the morbidity of allergies.

## Conclusion

The exact prevalence of food and drug allergies in Morocco is not known due to sparse and conflicting epidemiological data. There are many causes for the rise in FA. Beyond the now well acknowledged causes of the Western way of life, including the globalization of food and exposure to new allergens, more contemporary theories have been proposed instead (new foods, epigenetic modifications, genetic factors). In Morocco, few suspected drug hypersensitive people go through a full assessment to confirm a DA. Through the present investigation, we estimate that an awareness at the national level for clinicians to centralize the data would be very desirable to explain the dangerous fact of self-medication, and the negligence towards possible allergic events which can develop at any time, and which can inevitably lead to a manifestation that can be life-threatening for the person. The long-term objective would be to solve the problem of the care of affected patients as well as to sensitize pediatric clinicians, general practitioners and in particular the Moroccan population to this pathology. The concrete outcome of this preliminary investigation in our case would be to create a mandatory center and we may be able to increase our understanding of this subject and improve diagnosis and the management of patients by better understanding the frequency of these reactions and safe tests to determine a precise diagnosis. We can thus reduce morbidity, mortality and healthcare costs. For a better knowledge of FA and DA and their prevention and treatment in the future, it will be necessary to standardize terminology, advance our understanding of the pathophysiological mechanisms, and validate diagnoses using in vitro (Basophil Activation test and Lymphocyte Transformation test) and in vivo testing.

### What is known about this topic

- *Prevalence and Impact: food and drug allergies are prevalent in children, affecting around 8% of children in Western countries;*
- *Common allergens: Cow's milk, eggs, peanuts, tree nuts, soy, wheat, fish, and shellfish are common food allergens in children;*
- *Diagnosis and management: diagnosing food and drug allergies in children involves medical history, physical examination, and diagnostic tests such as skin prick tests, specific IgE blood tests, and oral food challenges*.

### What this study adds

- *For FA, the predominant allergen was cow's milk with 58.2% of the population. followed closely by egg allergy with a percentage of 23.4%;*
- *In FA, the symptomatic profile comprised the acute phase of the reaction, with acute urticaria being predominant accounting for 41.9% of the cases; in the case of DA, urticaria emerged as the most frequent manifestation, occurring in 48.3% of cases;*
- *The primary method for diagnosing FA was through prick tests, constituting 66% of the cases; in contrast, for DA diagnosis, allergological tests were less commonly employed*.
